# Getting to FP2020: Harnessing the private sector to increase modern contraceptive access and choice in Ethiopia, Nigeria, and DRC

**DOI:** 10.1371/journal.pone.0192522

**Published:** 2018-02-14

**Authors:** Christina Riley, Danielle Garfinkel, Katherine Thanel, Keith Esch, Endale Workalemahu, Jennifer Anyanti, Godéfroid Mpanya, Arsène Binanga, Jen Pope, Kim Longfield, Jane Bertrand, Bryan Shaw, on behalf of the FPwatch Group

**Affiliations:** 1 Population Services International, Washington, D.C., United States of America; 2 Population Services International–Ethiopia, Addis Ababa, Ethiopia; 3 Society for Family Health, Abuja, Nigeria; 4 Association de Santé Familiale, Kinshasa, Democratic Republic of Congo; 5 Tulane International, Kinshasa, Democratic Republic of Congo; 6 Independent consultant, Washington, D.C., United States of America; 7 Department of Global Health Systems and Development, Tulane University School of Public Health and Tropical Medicine, New Orleans, LA, United States of America; 8 Institute for Reproductive Health, Georgetown University, Washington, D.C., United States of America; National Academy of Medical Sciences, NEPAL

## Abstract

**Background:**

An estimated 214 million women have unmet need for family planning in developing regions. Improved utilization of the private sector is key to achieving universal access to a range of safe and effective modern contraceptive methods stipulated by FP2020 and SDG commitments. Until now, a lack of market data has limited understanding of the private sector’s role in increasing contraceptive coverage and choice.

**Methods:**

In 2015, the FPwatch Project conducted representative outlet surveys in Ethiopia, Nigeria, and DRC using a full census approach in selected administrative areas. Every public and private sector outlet with the potential to sell or distribute modern contraceptives was approached. In outlets with modern contraceptives, product audits and provider interviews assessed contraceptive market composition, availability, and price.

**Findings:**

Excluding general retailers, 96% of potential outlets in Ethiopia, 55% in Nigeria, and 41% in DRC had modern contraceptive methods available. In Ethiopia, 41% of modern contraceptive stocking outlets were in the private sector compared with approximately 80% in Nigeria and DRC where drug shops were dominant. Ninety-five percent of private sector outlets in Ethiopia had modern contraceptive methods available; 37% had three or more methods. In Nigeria and DRC, only 54% and 42% of private sector outlets stocked modern contraceptives with 5% and 4% stocking three or more methods, respectively. High prices in Nigeria and DRC create barriers to consumer access and choice.

**Discussion:**

There is a missed opportunity to provide modern contraception through the private sector, particularly drug shops. Subsidies and interventions, like social marketing and social franchising, could leverage the private sector’s role in increasing access to a range of contraceptives. Achieving global FP2020 commitments depends on the expansion of national contraceptive policies that promote greater partnership and cooperation with the private sector and improvement of decisions around funding streams of countries with large populations and high unmet need like Ethiopia, Nigeria, and DRC.

## Introduction

In developing regions, an estimated 214 million women have unmet need for family planning. Several global initiatives recognize the importance of modern contraceptive use, including the Sustainable Development Goals (2016–2030) which include universal access to family planning as one of nine sub-goals within health, and Family Planning 2020 (FP2020), a global commitment to provide an additional 120 million women and girls with universal access to a range of safe and effective modern contraceptive methods in the world’s least-developed countries.[1] The 2017 Family Planning Summit in London reasserted family planning as one of the ‘best buys’ in development, but recognized that most countries, are far from achieving their SDG and FP2020 goals and must accelerate progress.[2,3]

Engagement of the private sector is a critical strategy for accelerating progress towards these goals, [2,4] particularly in sub-Saharan African (SSA) countries with high unmet need and underserved geographies and populations. [5,6] More than one-third of all women currently using modern contraceptives in SSA report obtaining them through the private sector, the preferred source for many women. [6–9] Preference for the private sector is linked to perceptions of higher quality, specifically product availability, convenience, timeliness, and privacy. [8,10] Engagement of the private sector is often weak or strained and government policies can limit cooperation with the public sector. [11]

Achieving global SDGs and FP2020 commitments will depend on the success of countries with large populations and high unmet need. In SSA, key countries include Ethiopia, Nigeria, and Democratic Republic of Congo (DRC), each with a unique family planning policy environment and contraceptive method mix (see S1 Table). Ethiopia has made substantial financial investments in family planning and regulates public and private sector provision of methods and services. It has also managed to strengthen public-private engagement in the provision of injectables and long-acting reversible contraceptives (LARCs). [12,13] Social franchising has also increased provision of short-acting methods in the private sector. [14] Such initiatives have contributed to an increase in mCPR in Ethiopia and progress on FP2020 commitments. [15] In Nigeria [16] and DRC [17], large regional variations and inequalities in contraceptive access, lower use of LARCs, and a largely unregulated private sector have stymied progress toward national goals. Both countries have historically depended on condoms, short-acting methods, and traditional methods of family planning. [18,19] Recent initiatives have focused on private sector engagement through social franchising and mobile outreach service delivery, with pilot programs for LARC provision launched in 2015 in Nigeria[20,21] and DRC. [22] However, the current lack of formal policy and regulation as it relates to the private sector in each of these countries hinders the partnership and cooperation that is needed to strengthen public-private engagement. [16–18]

The field would benefit from systematically collected data on total contraceptive markets in the world’s least-developed countries, enabling policymakers’ to improve method diversity and choice. In this study, we measure contraceptive availability, market share, and price to highlight the potential of the private sector to increase access and meet client demand for short-acting methods and LARCs. We identify where national contraceptive policies could be expanded to promote greater partnership and cooperation with the private sector and decisions about funding streams strengthened to increase the role of the private sector in family planning provision.

## Methodology

The FPwatch Project, funded by the Bill & Melinda Gates Foundation, was designed to fill knowledge gaps in contraceptive markets by providing estimates for key family planning indicators. In SSA, this included Ethiopia, Nigeria and DRC. FPwatch surveys generate nationally- and regionally-representative data from a cross-section of outlets. These surveys complement other health facility surveys, such as the Performance, Monitoring and Accountability 2020 (PMA2020) surveys, [23] by providing a rigorous, robust, and in-depth look at contraceptive markets.

### Study design and sample selection

Data were collected in the second half of 2015. In Ethiopia, the geopolitical areas included the four most populous regions where 85% of the country’s population resides: Amhara, Oromia, Southern Nations, Nationalities, Peoples’ Region (SNNP) and Addis Ababa. The Nigerian survey was nationally representative: outlets from all six geopolitical zones were included. Two provinces were intentionally sampled in DRC to ensure the inclusion of one primarily urban and one primarily rural province, Kinshasa and Katanga, respectively. In 2015, DRC’s 11 original provinces were divided into 26. FPwatch used the pre-2015 boundaries in province selection.

Sample size requirements were based on estimates for the proportion of outlets with three or more modern methods of contraception in stock on the day of the survey at 95% confidence. This indicator was selected for its high relevance to FP2020 commitments (see S1 Table). One or two-stage sampling was conducted using Probability Proportional to Size in each study country, [24] with representative clusters of approximately 10,000 to 15,000 people. In DRC, a booster sample was included for public health facilities and pharmacies to adjust for insufficient numbers of these outlet types. A detailed sampling strategy is included in S2 and S3 Tables: Selected Clusters by Geopolitical Zones.

Data collection lasted six to eight weeks and field teams used a full census approach to identify potential outlets: data collectors met with local authorities to produce sketch maps of the areas’ potentially eligible outlets or walked down all streets and paths of selected administrative units to find them. Every public and private sector outlet with the potential to sell or distribute modern methods was screened for eligibility. Public sector outlets included hospitals, health centers, and community health workers and, in the case of DRC, representative private clinics in areas with no public facility. The private sector included private for-profit clinics, pharmacies, drug shops and general retailers, except in DRC where general retailers were not surveyed (Table 1).

**Table 1:** Outlet category description by country.

Outlet Category	Country		
	Ethiopia	Nigeria	DRC
**Public**	The public sector in Ethiopia includes public health facilities, and health posts staffed by health extension workers (HEWs). The public health facility category includes general and specialized national, regional and district hospitals as well as public health centers. HEWs are trained and paid women who deliver a package of basic health information and services to rural communities at stationary health posts. HEWs receive supportive supervision from health centers. Not-for profit outlets were included with public for analysis.	The Nigerian public sector consists of public health facilities and community health workers. Public health facilities include government (federal, state, local government area) facilities, like teaching hospitals and federal medical centers at the tertiary level; general hospitals at the secondary level; and primary health centers and clinics at the primary level. Community health workers include community-based health volunteers, like Community Health Extension Workers (CHEWs) and Role Model Mothers. Private not-for-profit health facilities include non-governmental (NGO) or mission/faith-based health facilities. Not-for profit outlets were included with public for analysis.	In DRC, the public sector consists of public health facilities and community health workers (CHWs). Public health facilities include (referral) hospitals, (referral) health centers, health posts, and dispensaries. This category also includes any private not-for-profit or for-profit health facilities that are designated by the government as the public health facility for the health area and equipped with a minimum basic package of services and commodities. Community health workers are community-based volunteers that are considered public sector outlets. CHWs in DRC can provide oral contraceptives, condoms and cycle beads. Not-for profit outlets were included with public for analysis.
**Private Clinics**	The private clinics category includes private hospitals and clinics. Clinics are tiered into lower, medium and higher clinics depending upon their size and the services they provide.	The private clinics category includes private hospitals and clinics.	The private clinics category includes Private (referral) hospitals, (referral) health centers, health posts and laboratories, run on a for-profit basis.
**Pharmacy**	The pharmacy category includes only nationally registered pharmacies. Pharmacies dispense medicines and compound prescribed preparations. Nationally registered pharmacists manage pharmacies.	Pharmacies are licensed by the government and are authorized to sell all classes of medicines including prescription-only medicines. Pharmacies are owned by registered pharmacists or owners employing the services of a registered pharmacist.	Pharmacies are licensed and regulated by the national medical authority and are staffed by pharmacists and qualified health practitioners. They sell all classes of medicine and are generally located in urban areas.
**Drug Shop**	The drug shop category consists of drug shops and rural drug vendors (RDVs). Pharmacists or druggists, with a diploma-level qualification, manage drug shops. While drug shops are registered and licensed, they are unable to compound prescribed preparations. Druggists, or pharmacy technicians, manage RDVs.	The drug shop category is comprised of Proprietary Patent Medicine Vendors (PPMVs). PPMVs are small-to-medium sized outlets selling primarily medicines. PPMVs may be registered nationally, however, many are not. PPMVs are legally permitted to sell over-the-counter medicines, including oral contraceptives.	Drug shops are smaller in size and scope than pharmacies. These facilities are not licensed by the national medical authority. They are sometimes owned or run by staff with primary health qualifications, such as nurses, but are most commonly run by staff with no health qualifications. They are ubiquitous in urban areas in DRC.
**General Retailer**	General retailers primarily consist of non-medicine shops and kiosks. General retailers are only legally allowed to provide condoms and do not typically have the staffing requirements to provide medicines, including contraceptive commodities other than condoms. Prior to FPwatch there was no data on the availability of contraceptives at these outlet types in Ethiopia.	The general retail category consists of supermarkets, mini-markets and kiosks primarily selling fast-moving consumer goods, food and provisions. Kiosks/tables are points of sale located in non-permanent structures that sell goods such as food, beverages and household items. Although retailers may have over-the-counter medicines including oral contraceptives available, national authorities do not regulate the sale of medicines by retailers. Prior to FPwatch there was no data on the availability of contraceptives at these outlet types in Nigeria.	*Not applicable*: *General retailers were not screened in DRC because these outlets do not stock contraceptives beyond condoms*.

Outlets must have met one of the following criteria to be eligible for a full interview and product audit: 1) stocked at least one modern contraceptive method other than condoms (oral contraceptives, emergency contraceptives, injectable contraceptives, contraceptive implants, or IUDs) on the day of the survey; 2) had one or more types of modern contraceptives other than condoms available within the three months preceding the survey; or 3) offered provider-dependent contraceptive services, including contraceptive injections, implant insertions, IUD insertions, and or male/female sterilization.

### Data collection

All eligible outlets, providers were invited to join the study after giving verbal informed consent. Data collectors used paper questionnaires to complete audits of relevant product information, including product brand name, generic name, active ingredient and corresponding strength, manufacturer name and country of manufacture. For each brand, providers/outlet staff reported on volume distributed during the previous month, stock out during the previous three months, and retail and wholesale price. At outlets where provider-dependent methods (IUDs, sterilizations, implants) were reportedly offered, a set of questions ascertained providers’ service readiness, including training, certifications and necessary equipment. Questionnaires were translated into local languages, reviewed by field teams and back-translated.

### Data analysis

Data were double entered into a Microsoft Access (Microsoft Corporation, Redmond, Washington, US) database by data entry personnel. All data analyses were conducted using Stata 14 and weighted using the inverse probability of cluster selection (StataCorp, College Station, Texas, USA).

A set of standard indicators was calculated for modern contraceptive methods, namely market composition, availability, stock-out, and median price. Modern contraceptive methods are defined as oral contraceptives, emergency contraceptives, contraceptive injections, contraceptive implants, IUDs, and male/female sterilization and do not include traditional methods. Availability is the percentage of all audited outlets that stocked at least one modern contraceptive method. Stock-out is when an outlet did not have a modern method on the day of the survey among outlets that said they offered the method in the past three months. Information on consumer price was collected in local currency. Price was then converted into USD using the average exchange rate during data collection and reported as cost per couple-years of protection (CYP). CYP is defined as the estimated protection a contraceptive provides over a one-year period. [25]

This study was approved by the National Research Ethics Review Committee at the Ministry of Science and Technology in Ethiopia, the National Health Research Ethics Committee of Nigeria and the Federal Ministry of Health, and the Comité D’Ethique, Ecole de Santé Publique, Université de Kinshasa in DRC.

#### Data sharing

All study data are available on the Harvard Dataverse as follows:

DRC: https://dataverse.harvard.edu/dataset.xhtml?persistendld=doi:10.7910/DVN/OJD10N#

Ethiopia: https://dataverse.harvard.edu/dataset.xhtml?persistendld=doi:10.7910/DVN/JRTCW5

Nigeria: https://dataverse.harvard.edu/dataset.xhtml?persistendld=doi:10.7910/DVN/2HRQON

#### Role of the funding source

The funders of the study had no role in study design, data collection, data analysis, data interpretation, or writing of the report. The corresponding author had full access to all the data in the study and had final responsibility for the decision to submit for publication.

## Results

### Potential market and actual market composition

**Fig 1:**
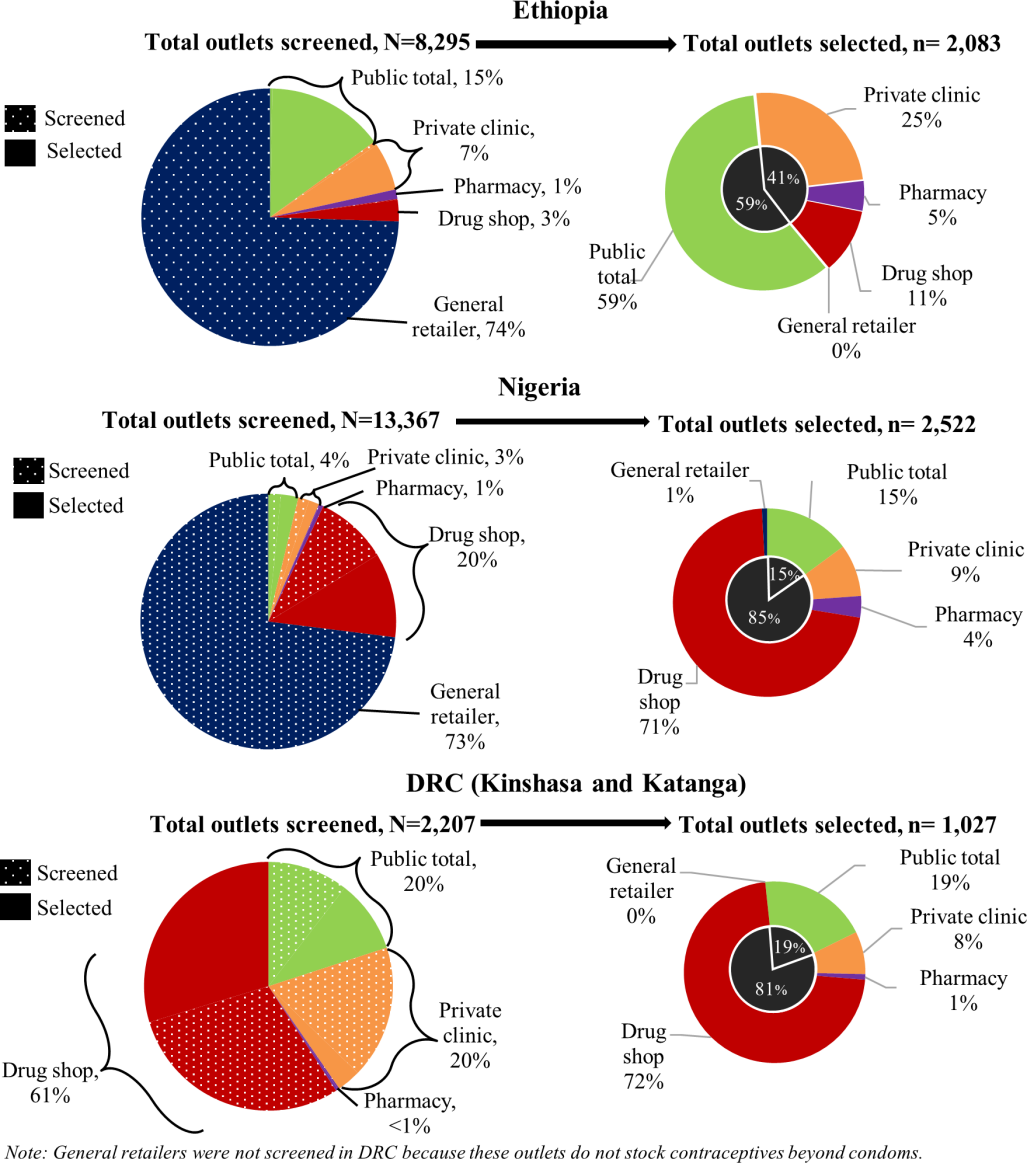
Total outlets screened (potential market) and eligible (actual market), by country and outlet type.

The potential market for modern contraceptive provision was dominated by the private sector: 85% of 8,295 screened outlets in Ethiopia including general retailers, 96% of 13,367 outlets in Nigeria including general retailers, and 80% of 2,207 outlets in DRC (Fig 1). General retailers were not screened in DRC because it was known at the time of the survey that these outlets did not stock contraceptives other than condoms. General retailers were screened in Ethiopia and Nigeria and made up approximately three-quarters of the potential market. However, they were excluded from subsequent analysis because they did not sell modern contraceptives other than condoms. Drug shops were the greatest proportion of private outlets screened in DRC (60%).

After excluding general retailers, 96% of all potential outlets in Ethiopia, 55% in Nigeria, and 41% in DRC had modern contraceptive methods on the day of the survey and made up the actual contraceptive market (solid pie slices on the left-hand side of Fig 1). In selected outlets in Ethiopia, the private sector was smaller than the public sector (41% vs. 59%); and private clinics (25%) were the largest group of private outlets. In Nigeria and DRC, private sector outlets were over 80% of the market with drug shops, also referred to as patent and proprietary medicine vendors (PPMVs) in Nigeria, making up 71% and 72% of the total actual market, respectively.

### Availability of any modern contraceptive method, excluding general retailers

Availability of any modern method other than condoms was similar within private and public outlets across countries. It was highest in Ethiopia (98% and 95% respectively) followed by Nigeria (59% and 54%), and DRC (47% and 42%). Among private outlets, pharmacies had the highest availability of modern contraceptives: 100% in Ethiopia, 89% in Nigeria, and 64% in DRC. Private clinics were least likely to have modern contraceptives available. Short-acting methods were more commonly available than LARCs or permanent methods (PMs), since only private clinics and public facilities were authorized to administer all methods. For a breakdown by outlet type see S4 Table.

### Availability of short-acting methods on the day of the survey

**Fig 2:**
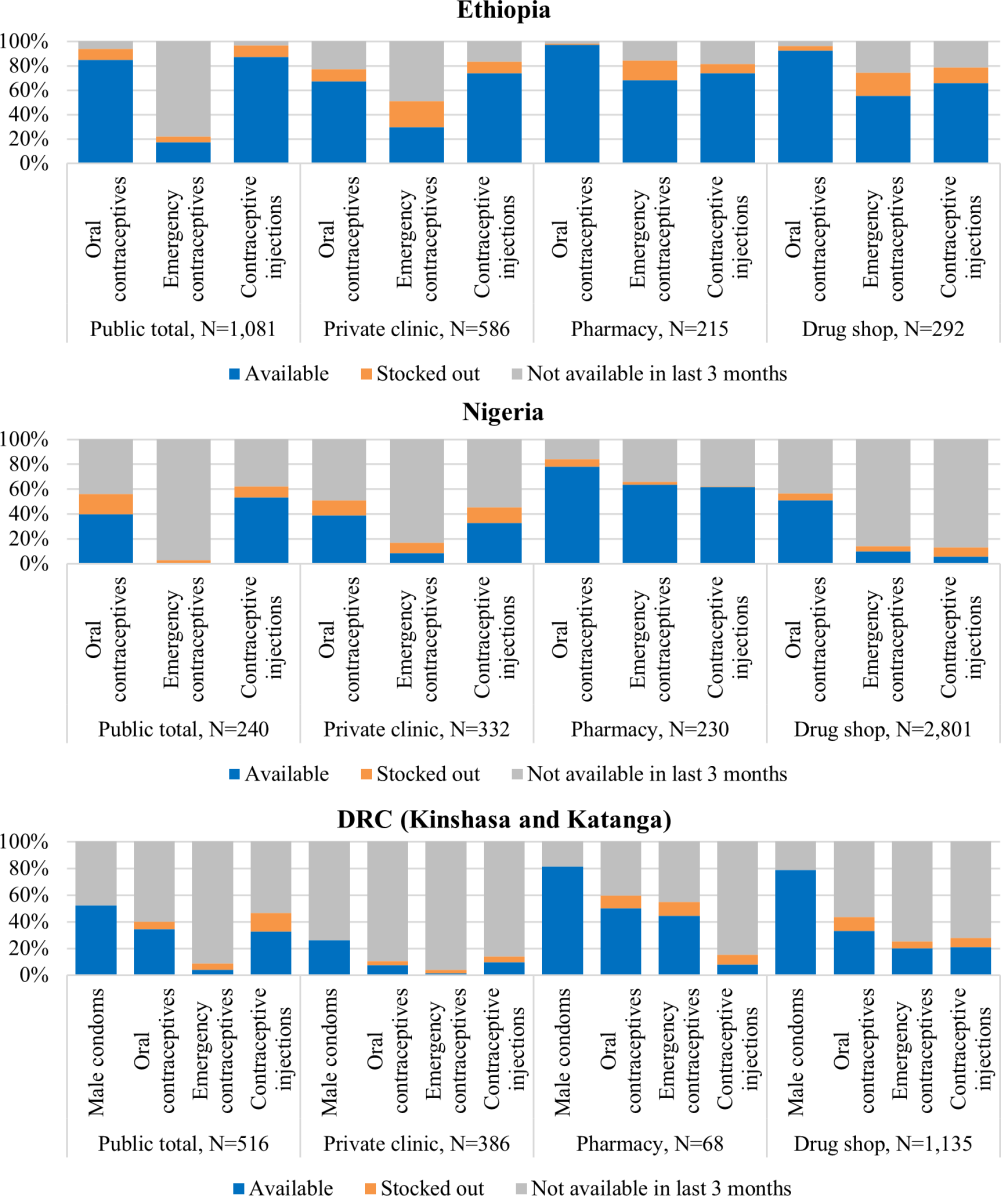
Availability of selected short-acting methods on the day of survey among all screened outlets excluding general retailers, by country and outlet type.

The private sector had higher availability of short-acting methods (oral contraceptives, emergency contraceptives, or injections) than the public sector in each country, particularly for emergency contraceptive pills (ECP). Ethiopia had the highest availability of short-acting methods in private sector outlets (56%-97% across methods, except for ECPs in private clinics 30%). In Nigeria, short-acting methods were more frequently available in pharmacies (61.6%-78.0%) than in other private sector outlets (8%-51%). In DRC, availability of all three short-term methods was less than 50% across the private sector (Fig 2). Stock-outs were observed in the private sector across all countries (1–21%).

In the private sector, pharmacies, followed by drug shops, had the highest availability of short-acting methods, particularly for commodities that do not require service provision. Oral contraceptive availability was highest in pharmacies in Ethiopia and Nigeria at 97% and 78%, respectively, and was just under half in DRC. The pattern was similar for oral contraceptives in drug shops. ECPs were concentrated in pharmacies, although availability was moderate: 68% in Ethiopia, 64% in Nigeria, and 44% in DRC. ECP availability was less than 20% in other private sector outlets in Nigeria and DRC as well as in all public sector outlets across all three countries.

### Availability of LARCs on the day of the survey

**Fig 3:**
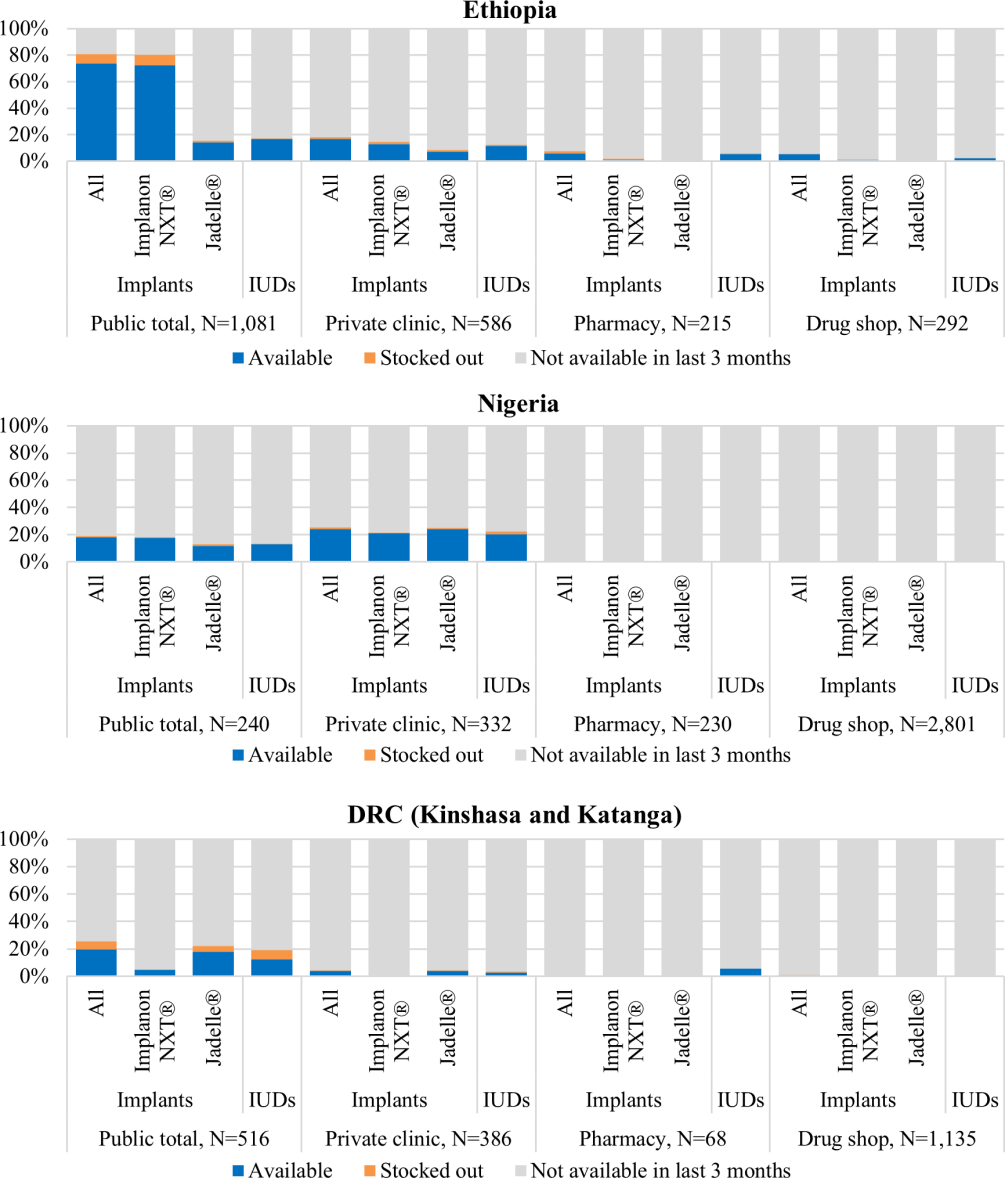
Availability of selected LARC methods on the day of the survey among all screened outlets excluding general retailers, by country and outlet type.

In the private sector, LARC availability (implants or IUDs) was low overall. When LARCs were available, they were found in private clinics where services can be rendered (3–24%) across all countries. Pharmacies and drug shops were not common outlets for LARCs, likely because they can only sell the commodity (<6%) (Fig 3). LARC availability in the public sector was higher in Ethiopia (17–74%) and DRC (13–20%) than their respective private sector counterparts. In Nigeria, LARC availability was slightly higher in private clinics (20–24%) than in the public sector (13–18%).

### Availability of choice: Three or more methods and three or more methods including LARC/PM on the day of the survey

**Fig 4:**
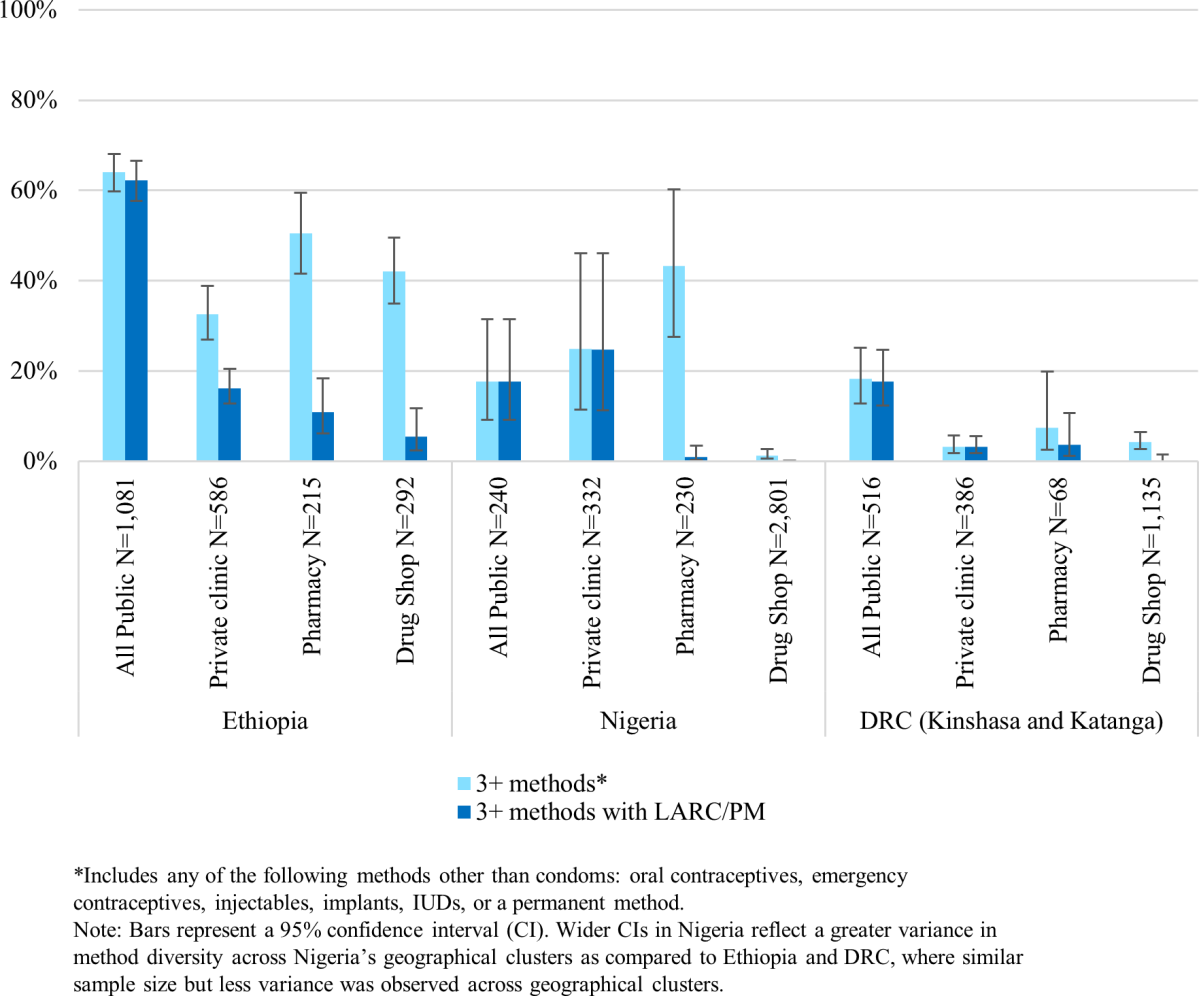
Percentage of outlets with 3+ methods and 3+ methods including LARC/PM on the day of the survey among all screened outlets excluding general retailers, by country and outlet type.

In Ethiopia and DRC, the private sector had lower availability of any three or more methods other than condoms (3+ methods) than the public sector. Conversely, in Nigeria, the private sector had greater method diversity than the public sector (Fig 4). Private sector outlets in Ethiopia and private clinics and pharmacies in Nigeria had moderate availability of 3+ methods (33–51% and 25%-43%, respectively) and were mostly limited to short-acting methods. Availability of 3+ methods in the private sector in DRC and drug shops in Nigeria was extremely low (<8%).

Private sector availability of 3+ methods including LARC/PM was low. The most common combination was oral contraceptives, injectables, and implants, and this pattern was fairly consistent across private sector outlet types. Private clinics in all countries had a more variable method mix that included permanent methods. Consistent with policy, no drug shops or pharmacies in any country had permanent methods.

### Price of modern contraceptives in the private sector

Contraceptive methods that are priced beyond an affordable range limit choice. All methods in Ethiopia were less expensive than in Nigeria and DRC. In Nigeria and DRC, prices per oral contraceptive and ECP dose were 25–166% higher in pharmacies than in drug shops. Prices per-dose for injectables were highest in private clinics across all countries, potentially due to the inclusion of an administration service fee (Table 2).

**Table 2:** Median consumer per-unit price of short-acting and LARC methods in USD in the private sector*, by country and outlet type.

Price in USD per dose	Private Clinics Average (n) [IQR]**	Pharmacies (n) [IQR]**	Drug Shops/PPMVs (n) [IQR]**
**Short-acting Methods**			
Oral Contraceptives			
Ethiopia	$ 0·15 (454) [0·15, 0·29]	$ 0·36 (494) [0·15, 0·58]	$ 0·15 (456) [0·15, 0·49]
Nigeria	$ 0·50 (76) [0·35, 0·50]	$ 0·50 (202) [0·25, 0·50]	$ 0·40 (1,643) [0·25, 0·50]
DRC	$ 0·33 (38) [0·00–0·55]	$ 0·88 (43) [0·40–3·41]	$ 0·33 (432) [0·22–0·55]
Emergency Contraceptives			
Ethiopia	$ 0·49 (170) [0·49, 0·73]	$ 0·49 (166) [0·49, 0·58]	$ 0·49 (189) [0·49, 0·58]
Nigeria	*$ 1·50 (6) [1·50*, *2·50]*	$ 1·25 (191) [0·25, 1·50]	$ 0·75 (449) [0·20, 1·25]
DRC	*$ 0·55 (8) [0·00–1·65]*	$ 1·65 (62) [1·10–5·50]	$ 1·32 (262) [1·10–1·65]
Injectables			
Ethiopia	$ 0·34 (415) [0·24, 0·49]	$ 0·24 (128) [0·24, 0·29]	$ 0·24 (172) [0·24, 0·39]
Nigeria	$ 2·50 (137) [2·00, 2·50]	$ 1·00 (217) [0·75, 1·50]	$ 1·00 (394) [0·75, 2·00]
DRC	$ 1·65 (49) [1·10–3·30]	*$ 0·55 (12) [0·44–0·55]*	$ 0·55 (243) [0·44–1·10]
**Long-acting Reversible Methods**			
Implants			
Ethiopia	$ 1·46 (180) [0·73, 1·95]	*$ 0·83 (8) [0·73*, *2·43]*	*$ 0·97 (10) [0·00*, *2·43]*
Nigeria	$ 7·50 (42) [5·00, 10·00]	*$ 6·00 (1) [6·00*, *6·00]*	*$ 2·50 (3) [2·50*, *2·50]*
DRC	$ 7·15 (24) [2·20, 10·12]	*$ —*	*$ 7·70 (7) [4·95*, *9·90]*
Implanon			
Ethiopia	$ 1·46 (84) [0·73, 2·43]	*$ 2·43 (1) [2·43*, *2·43]*	*$ —*
Nigeria	$ 5·00 (14) [5·00, 10·00]	*$ —*	*$ 2·50 (1) [2·50*, *2·50]*
DRC	*$ 10·12 (4) [0·00*, *10·12]*	*$ —*	*$ —*
Jadelle			
Ethiopia	$ 1·46 (49) [0·73, 1·95]	*$ 2·43 (2) [0·73*, *2·43]*	*$ —*
Nigeria	$ 7·50 (28) [7·50, 10·00]	*$ 6·00 (1) [6·00*, *6·00]*	*$ 2·50 (2) [2·50*, *2·50]*
DRC	$ 4·95 (20) [2·20, 11·00]	*$ —*	*$ 7·70 (7) [4·95*, *9·90]*
IUDs			
Ethiopia	$ 1·46 (84) [0·73, 2·43]	*$ 0·24 (5) [0·00*, *0·97]*	*$ —*
Nigeria	$ 5·00 (62) [5·00, 7·50]	*$ 1·25 (9) [1·25*, *1·25]*	*$ 0·80 (8) [0·75*, *1·00]*
DRC	*$ 4·95 (9) [3·30*, *5·06]*	*$ 66·22 (4) [8·25*, *67·64]*	*$ 2·20 (1) [2·20*, *2·20]*

*Majority of methods are free in the public sector and thus pricing is only presented for the private sector.

**Text in italics denotes strata with less than 15 observations.

It is important to consider cost-effectiveness in addition to the cost to consumer. Long-term use of each method changes its cost effectiveness; over time, LARCs are more cost effective. For example, in Nigerian drug shops, oral contraceptives had the lowest cost per unit ($0.40) versus injectables ($1.00) and implants ($2.50) that have higher per-unit costs. Over time, however, oral contraceptives were a less cost-effective means of family planning ($6.00 per CYP) compared to injectables ($4.50 per CYP) and implants ($0.83 per CYP), highlighting the need to overcome the high initial costs of LARCs. For a breakdown of price in USD per CYP by country and outlet type see S5 Table.

## Discussion

There is a missed opportunity in each country for modern contraceptive provision through the private sector. Low availability of methods and limited access to modern contraceptive stocking outlets make it difficult for women to adopt and use modern contraception consistently and plan the families they desire. Lack of access to a range of methods, short-acting, and LARC methods, and high prices limit choice and constrain a woman’s ability to make the most appropriate and cost-effective decision. In Ethiopia, short-acting methods are readily available in the private sector but LARC availability is more limited. The private sectors in Nigeria and DRC have low availability of both short-acting and LARC methods.

The ubiquity of drug shops within the potential market, but under-utilization in the actual market, present the greatest opportunity to increase access and expand choice in Nigeria and DRC. Although drug shops constitute nearly three-quarters of all outlets, half or less carry oral contraception, and even fewer carry other short-acting methods; almost none stock LARCs. Current evidence highlights the popularity of drug shops as preferred access points for young and unmarried women, youth, and underserved populations, [7,26,27] and supports leveraging these facilities to reach populations with unmet need. The higher availability of short-acting methods observed in pharmacies and drug shops compared to private clinics and the public sector suggests that they fill import gaps in the market.

Promoting access to an expanded range of short-acting methods has been consistently cited as a ‘high-impact practice’. [27] Unfortunately, there is currently little choice available within the private sector. Availability of oral contraceptives, ECPs, and injectables was low, particularly in Nigeria and DRC. Less than 10% of drug shops had three or more methods available in Nigeria and DRC. Lack of access to a preferred method is associated with inconsistent use or method discontinuation, which places women at greater risk for unintended pregnancy. [22] Short-acting methods require little-to-no service provision and are an appropriate fit for outlets without formally trained providers. Increasing short-acting method availability within drug shops would improve access and satisfy demand among discontinued or inconsistent users who prefer short-acting methods. Wider availability of short-acting methods is necessary to promote informed choice and is also particularly relevant for first-time users in contexts where either contraceptive information and counselling are available through trained providers but the supply is lower, or where provider training in drug shops can feasibly be facilitated.

High cost also limits choice. The price for ECPs was prohibitive in most private sector settings. Likewise, the high price of LARCs in Nigeria and DRC may present an obstacle to consumer access and choice. Making methods more widely available could create competition on the market and benefit consumers by decreasing high mark-ups caused by limited supply. Keeping costs down would also allow consumers to make more cost-effective choices. Subsidies across sectors, such as those in Ethiopia, likely offer the best short-term solution for reducing cost to consumers and increasing access to commodities with high-upfront costs such as LARCs.

In Ethiopia, a country that has a tightly regulated market and substantial financial commitments for family planning, pharmacies and drug shops have succeeded in providing affordable access to a variety of short-acting methods through strong social franchise networks. Pharmacies and drug shops have outperformed private clinics and the public sector, locations where greater emphasis has been placed on service provision as opposed to stocking self-administered commodities. [28] These outlets, through expanded provider training for counselling on both LARCs and short-acting methods, could also be leveraged to promote informed choice and affordable access to LARC commodities with referral to private clinics for services and counselling. Promoting market development through training and social franchising initiatives in the private sector are key strategies for reaching populations with unmet need with contraceptive methods and are showing promise for accelerating and sustaining FP2020 progress in SSA. [12,23,29,30]

Subsidized commodities have contributed to Ethiopia’s success in increasing access to LARCs among trained providers in the public sector and short-acting methods across sectors, and offer promise for other countries. Access to modern contraception at affordable prices could be offered through social marketing, including subsidizing methods with a service component, like LARCs. LARCs could be sold in drug shops and pharmacies with a referral for service delivery in private clinics with trained providers on LARC delivery. It is important, however, to incorporate new strategies to sustain access without over-relying on subsidies, like social business enterprise models that offer cross-subsidization of commodities.

Methods that are easy to administer, such as Sayana Press® injectables and Implanon NXT® implants, provide an opportunity to introduce service delivery for injectables and implants at private sector outlets. Social marketing projects, such as those piloted by Association de Santé Familiale DRC and Society for Family Health in Nigeria, that educate providers on the full range of methods already available and others that create demand for injectables and LARCs are promising for improved access to and utilization of a range of modern contraceptive methods in drug shops. [27,29,31,32]

Countries should adopt policies that permit drug shops to fill gaps in the public sector and private clinics. In Nigeria and DRC, private sector stakeholders are working with the public sector to improve the range of methods in drug shops and provider capacity for counselling and referral. [33] However, it is crucial to ensure that the regulatory environment can accommodate such measures. Steps taken by Tanzania’s Ministry of Health to incorporate drug shops into the private sector via targeted provider training and licensing as accredited drug dispensing outlets and other similar policy initiatives targeting drug shops in Uganda, can serve as models for Nigeria and DRC. [34,35]

### Strengths and limitations

To the best of our knowledge, no study has ever tracked contraceptive markets with such a large-scale, census-based approach as that used for FPwatch. We screened more than 25,000 outlets using a standardized survey format in Ethiopia, Nigeria, and DRC to understand contraceptive availability, market share, and price. We provide a comprehensive and current picture of where women are accessing LARCs and the potential of LARCs to help meet countries’ FP2020 commitments. The study had the following limitations: 1. Despite the large sample size, certain estimates by outlet type and strata resulted in small denominators; 2. Due to the point-in-time nature of data collection, we were not able to confidently capture data for mobile outlets. This may have underestimated contraceptive estimates for areas of DRC and Nigeria; 3. While price indicators are standardized, differing economic realities make direct comparisons by country and even assumptions about affordability within countries difficult to precisely evaluate. Additionally, the FPwatch project was designed to examine modern contraceptive commodity and service availability, market share, and price on the supply side. The project scope did not extend into measurement of consumer demand or behavior, or information on market process.

## Conclusion

There is a missed opportunity for modern contraceptive provision through the private sector, particularly in drug shops. Increasing choice and controlling price are essential for improving women’s access to and use of modern contraception. Strategies like social franchising, social marketing, and subsidizing products offer promise for opening up the private sector. Training providers and offering methods that are easier to administer are also important. National contraceptive policies should be expanded to promote greater partnership and cooperation with the private sector, including more strategic decisions about funding streams, to facilitate the role of the private sector in helping countries achieve their SDG and FP2020 commitments.

## Supporting information

S1 Table Modern contraceptive context within country. (PDF)

S2 Table Selected clusters by geopolitical zones in Nigeria and DRC (one-stage sampling). (PDF)

S3 Table Selected clusters by geopolitical zones in Ethiopia (two-stage sampling except for Addis Ababa). (PDF)

S4 Table Availability of modern contraceptive method types among all screened outlets, by country and outlet type. (PDF)

S5 Table Consumer price of short-acting and LARC methods in USD per CYP, by country and outlet type. (PDF)
