# Supplementary material for: Getting to FP2020: Harnessing the private sector to increase modern contraceptive access and choice in Ethiopia, Nigeria, and DRC
Source: PLoS One. 2018 Feb 14;13(2):e0192522. doi: 10.1371/journal.pone.0192522 (PMC5812628; doi:10.1371/journal.pone.0192522)
Supplement: S1 Table — (PDF) [file pone.0192522.s001.pdf]

Supplemental Table 1. Modern Contraceptive Context within Country:

	**Ethiopia**	**Nigeria**	**DRC**
Contraceptive measures* (MW= married women; UM= unmarried women)	-mCPR: 26·4%<br>-LARC mCPR: 9·9% MW, 11·5% UW<br><br>-Unmet need: 24·7%	-mCPR: 14·7%<br>-LARC mCPR: 1·5% MW, 1·4% UW<br><br>-Unmet need: 22·6 %	-mCPR: 10·0%<br>-LARC mCPR: ·9% MW, ·7% UW<br><br>-Unmet need: 40·8%
Current Method Mix	-LARCs: 15·2%<br>-Permanent: 0·4%<br>-Short-acting: 84·0%<br>-Other modern methods: 0·4%	-LARCs: 9·8%<br>-Permanent: 2·7%<br>-Short-acting: 82·2%<br>-Other modern methods: 5·4%	-LARCs: 7·6%<br>-Permanent: 6·3%<br>-Short-acting: 78·7%<br>-Other modern methods: 7·5%
FP2020 Commitments	-Increase contraceptive prevalence rate (CPR) to 55% by 2020<br>-Reduce total fertility rate (TFR) to 3 by 2020<br>-Reach an additional 6.2 million women and girls with family planning services<br>-Increase uptake of long-acting reversible methods<br>- Ensure commodities security,<br>-Scale up delivery of services for the hardest to reach groups	-Increase CPR by 2% every year to achieve 36% by 2018<br>- Strengthen the in-country logistics system that ensures commodity availability at the facility level<br>- Train at least 3,700 community health workers (CHWs) to deliver the range of contraceptives, particularly long-acting and reversible methods and support task shifting so CHWs in rural areas can provide multiple methods.	-Increase the CPR from 5·4% in 2010 to 19% in 2020.<br>- Increase the number of users of modern methods from 700,000 in 2010 to 2·1 million in 2020<br>- Provide an increased range of at least 3 contraceptive methods in health facilities offering family planning: the extended range of contraceptive methods with at least 1 long-acting method

* The data for mCPR and unmet need were reported in the 2016 FP2020 Progress Report and were produced using the Track20 FPET model. The data for LARC mCPR and current method mix are from DHS reports in Ethiopia (2016), Nigeria (2013) and DRC (2013-14).
