# Supplementary material for: Getting to FP2020: Harnessing the private sector to increase modern contraceptive access and choice in Ethiopia, Nigeria, and DRC
Source: PLoS One. 2018 Feb 14;13(2):e0192522. doi: 10.1371/journal.pone.0192522 (PMC5812628; doi:10.1371/journal.pone.0192522)
Supplement: S2 Table — (PDF) [file pone.0192522.s002.pdf]

Supplemental Table 2: Selected Clusters by Geopolitical Zones in Nigeria and DRC (One-stage sampling)

Country	Geopolitical Zones (Strata)	First Stage Selected Clusters
Nigeria		Localities
	North Central	58
	North East	25
	North West	29
	South East	39
	South South	30
	South West	19
DRC		Health Areas
	Kinshasa	34
	Katanga	80
