# Supplementary material for: Getting to FP2020: Harnessing the private sector to increase modern contraceptive access and choice in Ethiopia, Nigeria, and DRC
Source: PLoS One. 2018 Feb 14;13(2):e0192522. doi: 10.1371/journal.pone.0192522 (PMC5812628; doi:10.1371/journal.pone.0192522)
Supplement: S3 Table — (PDF) [file pone.0192522.s003.pdf]

Supplemental Table 3: Selected Clusters by Geopolitical Zones in Ethiopia (Two-stage sampling except for Addis Ababa)			
**Country**	**Geopolitical Zones (Strata)**	**First Stage Selected Clusters**	**Second Stage Selected Clusters**
		Woredas	Kebeles
**Ethiopia**	Addis Ababa	N/A*	27
	Amhara	30	300
	Oromia	30	300
	SNNPR	33	330
* Addis Ababa does not have woredas so a single stage sample was taken at the level of the kebele using PPS.			
