# Supplementary material for: Getting to FP2020: Harnessing the private sector to increase modern contraceptive access and choice in Ethiopia, Nigeria, and DRC
Source: PLoS One. 2018 Feb 14;13(2):e0192522. doi: 10.1371/journal.pone.0192522 (PMC5812628; doi:10.1371/journal.pone.0192522)
Supplement: S4 Table — (PDF) [file pone.0192522.s004.pdf]

Supplemental Table 4: Availability of modern contraceptive method types among all screened outlets, by country and outlet type				
**Availability of Methods, Excluding condoms**	All Public	Private for profit	Pharmacy	Drug Shop
**Ethiopia**	***n=1,081***	***n=586***	***n=215***	***n=292***
Any modern method (exc. condoms)	97·8	93·0	100·0	95·7
Any short-acting methods (exc. condoms)	96·1	83·2	100·0	95·7
Any LAPM	74·1	18·4	10·9	6·5
**Nigeria**	***n=240***	***n=332***	***n=230***	***n=2801***
Any modern method (exc. condoms)	59·1	49·7	88·5	53·7
Any short-acting methods (exc. condoms)	56·1	46·6	88·5	53·7
Any LAPM	21·7	30·2	1·2	0·0
**DRC**	***n=516***	***n=386***	***n=68***	***n=1135***
Any modern method (exc. condoms)	47·3	15·4	64·4	50·1
Any short-acting methods (exc. condoms)	42·9	14·1	64·4	50·0
Any LAPM	24·1	5·7	5·5	0·6
