# Supplementary material for: Getting to FP2020: Harnessing the private sector to increase modern contraceptive access and choice in Ethiopia, Nigeria, and DRC
Source: PLoS One. 2018 Feb 14;13(2):e0192522. doi: 10.1371/journal.pone.0192522 (PMC5812628; doi:10.1371/journal.pone.0192522)
Supplement: S5 Table — (PDF) [file pone.0192522.s005.pdf]

Supplemental Table 5: Consumer price of short-acting and LARC methods in USD per CYP, by country and outlet type

**Price in USD per CYP**	Private for-profit	Pharmacies	Drug Shops/PPMVs
Oral Contraceptives			
Ethiopia	$ 2.19 (454) [2.19, 4.38]	$ 5.47 (494) [2.19, 8.76	$ 2.19 (456) [2.19, 7.30]
Nigeria	$ 7.50 (76) [5.25, 7.50]	$ 7.50 (202) [3.75, 7.50]	$ 6.00 (1,643) [3.75, 7.50]
DRC	$ 4.95 (38) [0.00-8.25]	$ 13.20 (43) [6.05-51.15]	$ 4.95 (432) [3.30-8.25]
Emergency Contraceptives			
Ethiopia	$ 9.73 (170) [9.73, 14.60]	$ 9.73 (166) [9.73, 11.68]	$ 9.73 (189) [9.73, 11.68]
Nigeria	$ 30.00 (6) [30.00, 50.00]	$ 25.00 (191) [5.00, 30.00]	$ 15.00 (449) [4.00, 25.00]
DRC	$ 11.00 (8) [0.00-33.00]	$ 33.00 (62) [22.00-110.00]	$ 26.40 (262) [22.00-33.00]
Injectables			
Ethiopia	$ 1.36 (415) [0.97, 1.95]	$ 0.97 (128) [0.97, 1.17]	$ 0.97 (172) [0.97, 1.56]
Nigeria	$ 10.00 (137) [10.00, 15.00]	$ 4.50 (217) [3.00, 6.00]	$ 4.50 (394) [4.00, 8.00]
DRC	$ 6.60 (49) [4.40-13.20]	$ 2.20 (12) [1.76-2.20]	$ 2.20 (243) [1.76-4.40]
Implants			
Ethiopia	$ 0.46 (180) [0.29, 0.64]	$ 0.26 (8) [0.19, 0.84]	$ 0.30 (10) [0.00, 0.76]
Nigeria	$ 2.00 (28) [1.97, 2.63]	$ 1.58 (1) [1.58, 1.58]	$ 0.83 (2) [0.66, 1.00]
DRC	$ 1.88 (24) [0.58-3.91]	$ --	$ 2.03 (7) [1.30-2.61]
IUDs			
Ethiopia	$ 0.32 (84) [0.16, 0.53]	$ 0.05 (5) [0.00, 0.21]	$ --
Nigeria	$ 1.00 (47) [1.09, 1.63]	$ 0.27 (7) [0.27, 0.27]	$ 0.17 (6) [0.16, 0.22]
DRC	$ 1.08 (9) [0.72-1.10]	$ 14.40 (4) [1.79-14.70]	$ 0.48 (1) [0.48-0.48]
